# Multi-variant differential evolution algorithm for feature selection

**DOI:** 10.1038/s41598-020-74228-0

**Published:** 2020-10-14

**Authors:** Somaia Hassan, Ashraf M. Hemeida, Salem Alkhalaf, Al-Attar Mohamed, Tomonobu Senjyu

**Affiliations:** 1 Electrical Engineering Department, Faculty of Engineering, Aswan University, Aswan, Egypt; 2 Electrical Engineering Department, Faculty of Energy Engineering, Aswan University, Aswan, 81528 Egypt; 3 Department of Computer, College of Science and Arts in Ar-Rass, Qassim University, Ar Rass, Saudi Arabia; 4 Department of Electrical and Electronics Engineering, Faculty of Engineering, University of the Ryukyus, Nishihara, Japan

**Keywords:** Engineering, Mathematics and computing

## Abstract

This work introduces a new population-based stochastic search technique, named multi-variant differential evolution (MVDE) algorithm for solving fifteen well-known real world problems from UCI repository and compared to four popular optimization methods. The MVDE proposes a new self-adaptive scaling factor based on cosine and logistic distributions as an almost factor-free optimization technique. For more updated chances, this factor is binary-mapped by incorporating an adaptive crossover operator. During the evolution, both greedy and less-greedy variants are managed by adjusting and incorporating the binary scaling factor and elite identification mechanism into a new multi-mutation crossover process through a number of sequentially evolutionary phases. Feature selection decreases the number of features by eliminating irrelevant or misleading, noisy and redundant data which can accelerate the process of classification. In this paper, a new feature selection algorithm based on the MVDE method and artificial neural network is presented which enabled MVDE to get a combination features’ set, accelerate the accuracy of the classification, and optimize both the structure and weights of Artificial Neural Network (ANN) simultaneously. The experimental results show the encouraging behavior of the proposed algorithm in terms of the classification accuracies and optimal number of feature selection.

## Introduction

Artificial Neural Network (ANN) have been widely used in scientific problems and have attracted many researchers as the most popular tool for pattern classification, regression, and recognition due to its nonlinearity. The most challenging matter in ANN models is the selection of the appropriate weights, number of layers, and number of nodes in each layer. The complexity of the network is affected by the number of layers and nodes, so the difficulty for the training process will be increased. Therefore, selecting the suitable ANN model is required which should not be very small network that has a limited potential to be able to characterize the real state nor a large network which doing complex training process and may provide noise in the training data and thus cannot represent superior capability^1–4^.

Feature selection (FS) supplies a way to reduce the number of features from a large number of available features to capture better classification performance than using all features by removing or reducing irrelevant and redundant features^5,6^. A dataset usually includes a large number of features in classification problems, so irrelevant and redundant data are not applicable for classification and they may reduce the performance of the classification due to the large search space. FS strategies can be divided generally into three categories: filter, wrapper, and hybrid techniques. Filter technique dependently operates on data itself using appointed methods such as Principal Component Analysis (PCA) which is a common method. On the other hand, wrapper techniques are beneficial in finding feature subsets that satisfy a predetermined classifier. Consequently, they are broadly examined for the accuracy of the class. Besides that wrapper approaches are expensive and can be collapsed with a very large number of features due to utilizing learning algorithms in evaluating feature subset every time. However, hybrid techniques try to gather merits of the filter and wrapper techniques by manipulating their correlative strengths^7,8^. FS plays very important role in many areas such as pattern classification, multimedia information retrieval, data mining, machine learning applications and so on, which can influence the classification accuracy rate and reducing the time required for training. Classifying any given input feature vector into pre-defined set of classes of patterns needs assigning this vector to one of a set of classes^9,10^.

Meta-heuristics have been very dependable for solving diverse optimization problems in the last two decades and overcoming the challenging problem of searching optimal subset from all the original set. A new FS approaches were generated based on evolutionary optimization techniques since they can lead to a faster way to find optimal solutions. Moreover, by considering an effective fitness function a high dimensional data can be managed by limited number training samples. Whereas the complete search generates all possible solutions for the problem, meta-heuristics present outstanding performance compared to other conventional search techniques^11–13^.

## Background

### Related works

In recent years, several meta-heuristics have been utilized by many researchers in the field of optimization to search feature subset space for selecting optimal feature set. The strategy of meta-heuristic may determine a satisfactory solution in a reasonable time in spite of it doesn’t assure finding the best solution in every run. These algorithms showed superior performance in solving many practical problems which can be original, modified, or hybrid algorithms^14–20^. In^21^, Menghour and Meslati introduced a hybrid feature selection algorithm based on Ant Colony Optimization (ACO) and Particle Swarm Optimization (PSO) algorithms. The algorithm was designed to solve six well-known datasets. Artificial Bee Colony optimization (ABC) and DE technique was proposed in^22^ as a new combinable method for feature selection of classification tasks for solving fifteen public datasets. In^23^, a new hybrid ACO-ABC algorithm was introduced to validate thirteen datasets’ problems, in which ants decide the best ant and best feature subset by exploiting the bees and adjusting as their sources of food. In addition, a feature selection approach was proposed based on two different implementation of multi-objective ABC algorithm combined with non-dominated sorting procedure and genetic operators for examining twelve benchmark datasets^24^. In^25^, a new method called PSO-DFS using bare-bone particle swarm optimization (BBPSO) for discretization and feature selection in a single stage was proposed for solving ten high-dimensional datasets. In^26^, a comprehensive study to investigate the use of Genetic programming (GP) for feature construction and selection on high-dimensional classification problems was presented and tested on seven high-dimensional gene expression problems. Chen et al. proposed two novel Bacterial Foraging Optimization algorithms (BFO), which named Adaptive Chemotaxis Bacterial Foraging Optimization algorithm (ACBFO) and Improved Swarming and Elimination-Dispersal Bacterial Foraging Optimization algorithm (ISEDBFO) to create the mapping relationship between the bacterium and the feature subset and to evaluate the importance of features. This method dealt with feature selection problems and tested ten public datasets of UCI^27^. Majdi et al. proposed a Grasshopper Optimization Algorithm (GOA) as a search strategy to design a wrapper-based feature selection method in the form of four different strategies to moderate the immature convergence and stagnation drawbacks of the conventional GOA. These approaches were benchmarked on twenty-two public UCI datasets^28^.

In^29^, the antlion multi-objective wrapper-based feature selection method (CALO) was proposed by using different chaotic Maps and tested on eighteen datasets to balance between exploration and exploitation in the search space. The proposed method achieved better performance by converging to the optimal solution than PSO and GA methods and it was more effective than the original ALO method.

Li et al. introduced a new multi-objective ranking binary artificial bee colony method for the gene selection on eight microarray datasets. They first used the Fisher Markov Selector method to assort and choose the features that will be used as inputs to the binary ranking artificial bee colony. After that, the binary ranking artificial bee colony selected the genes subset. This method achieved the best performance compared to other methods with different classifiers. The results show also outperforms of that method in selecting smaller number of selected features^30^.

In spite of the advantages of the above mentioned heuristic algorithms for feature selection on classification problems, someone may inquire if we need another new heuristic algorithms. The theory of No-Free-Lunch (NFL) illustrated that all the optimization problems cannot be solved by one optimizer^31^. Thus, all classification/ feature selection problems cannot be solved by only one of the heuristic feature selection methods and there is always a possibility to improve the current methods to solve better the current new classification/feature selection problems. This is our motivation for attempting to propose another optimization algorithm for feature selection on classification.

### Cat Swarm Optimization (CSO)

Cat Swarm Optimization algorithm was proposed in 2007 by Chu and Tsai^32^. The CSO algorithm has two modes: seeking mode and tracing mode. In the beginning of the iteration, the number of cats is specified and cats broadcast arbitrarily in M-dimensions space. Then, applying cats to solve the problem, in which every cat has position, velocity for each dimension, fitness value, and a flag to determine if the cat in seeking or tracing mode. The one of the cats with the last solution will have the finest position. At the end of the iterations, the best solution will be kept^33^.

### Whale Optimization Algorithm (WOA)

Whale Optimization Algorithm was presented by Mirjalili and Lewis in 2016^34^.This algorithm comprises of two main stages; encircling prey and spiral updating position in the first stage (exploitation stage). In the second stage, a random searching for a prey is carried out (exploration stage). In the beginning, whales are allocated by arbitrary solutions and the minimum or maximum value of the objective function will be assumed as the best optimal value relying on the problem is solved. Then, every search agent of the objective function is calculated. Every search agent modifies its position relying on the best solution or on a random choice search agent for every iteration.

### Sine Cosine Algorithm (SCA)

Sine Cosine Algorithm was proposed in 2015 by Mirjalili^35^.In SCA, the algorithm started by arbitrary solutions’ set. The objective function calculated recurrently this arbitrary set and rules’ set which is the core of this method was used to improve it. It consists of two phases: in the first phase (exploration phase), the arbitrary solutions in the solutions’ set were combined suddenly by the optimization method to find the encouraging search space areas. Random solutions were changed gradually in the exploitation phase. In addition, arbitrary differences were greatly fewer than those in the first phase.

### Differential Evolution Algorithm (DE)

Differential Evolution (DE) algorithm was introduced by Storn and Price in 1996^36^. It is one of the most popular evolutionary algorithms to solve the global optimization problems. Global optimization is necessary in fields such as engineering, statistics and finance. It is stochastic and population-based optimization algorithm. It is developed to optimize real valued functions and real parameter. A population of candidate solutions for the optimization problem to be solved is randomly initialized. By applying crossover and mutation, new individuals are created for each generation of the evolution process. Recombination of the target individual with mutant individual to create the trial individual incorporates successful solutions from the previous generation. The target individual is compared with the trial individual and the one with the lowest function value is admitted to the next generation. Mutation, recombination and selection continue until some stopping criterion is reached^37,38^.

The mutation is performed by computing the vector differences between other two individuals in the same population which are selected randomly. Generating the mutant individual $V_{i,g}$ by adding the weighted difference of two of the vectors $F\left( {X_{{r_{2,g} }} + X_{{r_{3,g} }} } \right)$ to the base vector *X*_*r1*, g_ to disorganize it. A mutant vector is generated by the following formula:

1 $$ V_{i,g} = X_{{r_{1,g} }} + F\left( {X_{{r_{2,g} }} + X_{{r_{3,g} }} } \right) $$

where *r*_*1*_, *r*_*2*_ and *r*_*3*_ are indexes selected randomly over [*1, N*], *N* is the number of individuals in the population, *g* is the current generation, and *F* is a constant mutation factor from [0, 2].

The trial vector ${\text{U}}_{{{\text{i}},{\text{g}}}}$ is constructed through the recombination step where is developed from the elements of the target vector, ${\text{X}}_{{{\text{i}},{\text{g}}}}$, and the elements of the mutant vector, $V_{i,g}$*.* The crossover factor ${\text{CR}}$ presents the probability of entering elements of the mutant vector with the trial vector. The trial vector constructed formula:

2 $$ {\text{U}}_{{{\text{i}},{\text{j}},{\text{g}}}} = \left\{ {\begin{array}{*{20}l} {{\text{V}}_{{{\text{i}},{\text{j}},{\text{g }}}} } \hfill & {\quad if\,{\text{rand }}_{{{\text{i}},{\text{j}}}} \le CR\,or\,J = {\text{J}}_{{{\text{rand}}}} } \hfill \\ {{\text{X}}_{{{\text{i}},{\text{j}},{\text{g }}}} } \hfill & {\quad if\,{\text{rand }}_{{{\text{i}},{\text{j}}}} > CR\,and\,J \ne {\text{J}}_{{{\text{rand}}}} } \hfill \\ \end{array} } \right. $$

where *i* = *1, 2. . . N*; *N* is the population size, *j* = *1, 2. . . D; D* is the dimension of a single vector. ${\text{rand }}_{{{\text{i}},{\text{j}}}}$ is a random number in range [0, 1] and ${\text{J}}_{{{\text{rand}}}}$ is a random integer from [1, 2, …,D].

Finally, the trial vector ${\text{U }}_{{{\text{i}},{\text{g}}}}$ is compared to the target vector ${\text{X}}_{{{\text{i}},{\text{g}}}}$ and the one with the lowest function value is become a member of the next generation *g* + *1* using the fitness function formula:

3 $$ {\text{X}}_{{{\text{i}},{\text{g}} + 1}} = \left\{ {\begin{array}{*{20}l} {{\text{U}}_{{{\text{i}},{\text{j}},{\text{g }}}} } \hfill & {\quad if\,f\left( {{\text{U }}_{{{\text{i}},{\text{g}}}} } \right) < f\left( {{\text{X}}_{{{\text{i}},{\text{g}}}} } \right)} \hfill \\ {{\text{X}}_{{{\text{i}},{\text{g }}}} } \hfill & {\quad otherwise} \hfill \\ \end{array} } \right. $$

Recently, DE has arisen as an encouraging approach in several real world challenges. Effectiveness, robustness, capability to deal with complex large-dimensional optimization problems, and needing few control parameters are some merits of DE algorithm over other meta-heuristic algorithms. Furthermore, because of the fitness of offspring is competed and compared one-to-one with the fitness of corresponding parent, DE has sufficiently fast convergence characteristics. Although this approach raises the possibility of trapping in local optimal (suboptimal solution) and leads to premature convergence, it may be efficient to find an optimal solution rapidly. The control parameters of DE are needed a fine-tuning while remain fixed over the process of optimization is considering another demerit of DE.

To overcome the drawbacks of DE algorithm, it has been integrated with other optimization algorithms in a hybridized form to improve the performance of DE. On contrast, it is an effective method on a large range of classic optimization problems. DE is one of the most popular heuristic algorithms to solve single-objective optimization problems and it has been extended to solve multi-objective optimization problems^39^.

A novel optimization algorithm is proposed in this work to optimize both the weights and the structure of ANN simultaneously by presenting a new solution representation. Two main phases are composed of the proposed method: arrangement optimization and weights update. However, the proposed MVDE algorithm with multi variant mutation and adaptive scaling factor has been developed to choose the optimal number of features used for classification which has an impact on the accuracy of the classification.

However, the proposed MVDE is presented to overcome the drawbacks of DE which mentioned above. On this issue, different five mutation approaches incorporated with two high random scaling factors based on cosine and logistic distributions will be used to maintain the population diversity during the optimization process which leads to preventing premature convergence. Moreover, the requirements for tuning of the control parameters can be reduced by the proposed adaptive crossover and adaptive selection.

In addition, the experimental results are compared to the results in the literature and to another four optimization algorithms; DE, CSO^32,33^, WOA^34^, SCA^35^, and PSO^21^ for evaluating the proposed algorithm performance.

The rest of this paper is organized as follows: The methodology of the proposed approach is outlined in "Methodology" section. "Results and discussion" introduces and analyses the experimental results. Finally, conclusions is given in "Conclusion" section.

## Methodology

### Proposed multi-variant differential evolution algorithm

**Figure 1:**
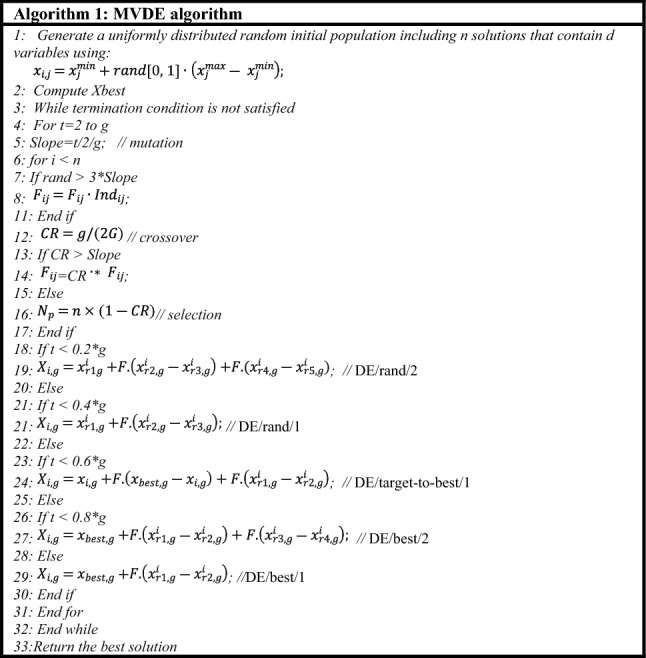
Pseudo code of MVDE method.

Differential evolution (DE) algorithm^36^ is a promising tool that can be regarded to deal with complicated high-dimensional problems with enhanced search qualities. DE algorithm is selected here to use as a search engine because it has meaningful merits over other meta-heuristic methods in terms of robustness, effectiveness, and fast convergence characteristics in searching high-scale problems. On contrast, the premature convergence to local optima and fixed control parameters are the problems of DE^37–39^. Additional improvements are essential before using this method in practical problems to gain better performance. In this concern, a new algorithm named Multi-Variant Differential Evolution (MVDE) has been proposed as an almost parameter-free optimization method. The proposed approach was designed mainly to enhance the global search ability of the original DE, i.e., reducing the probability of trapping in local optima and preventing the premature convergence. The proposed MVDE uses five different mutation strategies integrated with two high random scaling factors (based on cosine and logistic distributions) to maintain the population diversity during the optimization process, thereby prevent premature convergence. Additionally, the proposed adaptive crossover integrated with adaptive selection to reduce the needs for tuning of the control parameters. The overall steps for proposed MVDE algorithm are shown in Fig. 1. The main stages of MVDE algorithm are listed below.

#### Initialization

The proposed MVDE is designed as population-based meta-heuristic algorithm. It begins to solve d-dimensional optimization problem with initial solutions ($x_{j,i,0}$, initial candidate population) produced randomly to overspread the restricted search space as best as possible as follows:

4 $$ x_{i,j} \left( 0 \right) = x_{j}^{min} + rand\left[ {0, 1} \right] \cdot \left( {x_{j}^{max} - x_{j}^{min} } \right)\quad \forall i \in \left\{ {1,2, \ldots ,n} \right\}, j \in \left\{ {1,2, \ldots ,d} \right\} $$

where, $n$ is the population size, $x_{j}^{min}$ and $x_{j}^{max}$ are the minimum and maximum limits of the ${\text{j}}$-dimensional, and $rand\left[ {0, 1} \right]$ is a uniformly distributed random variable between 0 and 1 respectively.

In the next generation, MVDE goes in a cycle of iteration using a new single operation named multi-variant mutation-crossover process in order to create a new population. This process is associated with two proposed operators named self-adaptive scaling factor (incorporated with two different distributions and adaptive crossover operator) and adaptive parent selection. In the beginning, it is essential to explain the mutation-crossover process by describing its associated operators.

#### Self-adaptive scaling factor

Scaling factor ${\text{F}}$ has substantial effect on the convergence speed as a favorable control parameter. With the progress of iterations, the speed of convergence can be generally enhanced by reducing gradually ${\text{ F}}$. In^40^, a self-adaptive scaling factor of DE was introduced based on the elitist scheme of the learning rate to keep track of the fittest vector (i.e., the best individual is copied into the next generation). In spite of the widespread use of elitist scheme in GAs for attempting to help for fast convergence, it might be suffering from the problem of premature convergence. In^41^, biological genetic strategy has been proposed to inspire another adaptive scaling factor by changing (increasing or decreasing) F exponentially between pre-specified initial and maximum scaling factors besides to two of adjustable elements. However, extra parameters have been involved by this adaptive scaling factor which need to be chosen or adjusted.

In this work, to enhance the adaptive and dynamic performance of MVDE, a new adaptive scaling factor is proposed. The values of random variables which are generated from two different probability distributions: cosine and logistic distributions are used to update the scale factor of each individual for each generation.

##### Proposed distributions

The two parametric distributions with different thicknesses tails which can be identified completely are cosine and logistic distributions. Depending on the development of optimization operation, the probability of selecting each random generator is proposed.

In the first generations, F has a value with a high probability to be produced according to the cosine distribution. On the other hand, at the end of optimization, the probability of utilizing the logistic distribution is increased. Hence, both of these distributions are used simultaneously. Certainly, small and large values of independent variables can be generated from each random distribution (i.e., symmetrical bell shaped distributions). Therefore, the control of the exploration (with large values) and the exploitation (with small values) are attempted to be supported by this proposed scheme for controlling the exploration– exploitation balance.

**Figure 2:**
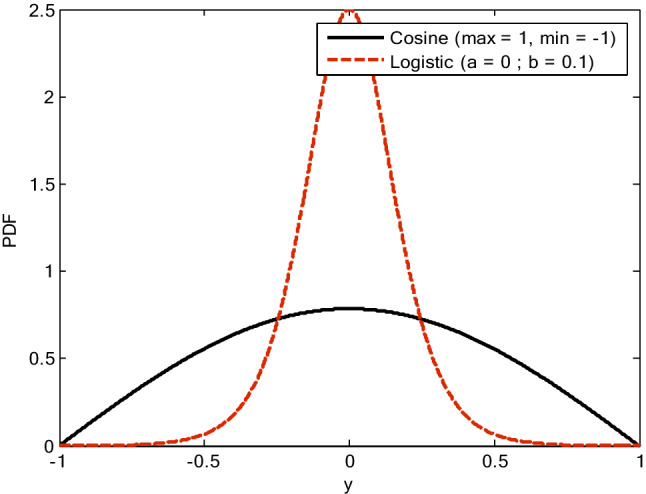
The distributions of cosine and logistic.

As shown in Fig. 2, the logistic distribution has a lighter tail than the cosine distribution. Therefore, sufficient disturbances are obtained by applying the heavy tail cosine distribution for spreading the population over the wide search space in the beginning of optimization operation. The cosine distribution is utilized instead of the normal distribution due to its qualified modeling the higher proportion of the heavy tail of the population for diversity conservation, which will avoid premature convergence. However, the lighter tail logistic distribution is used at the end of the optimization process to increase the exploitation ability. Therefore, the proposed adaptations of the scale factor give the meaningful advantage for solving the exploration–exploitation problem during the complete optimization process. In^41^, the proposed two types of distributions that are used by the proposed MVDE have been introduced.

###### The cosine distribution

The cosine or half-cosine distribution is able to transact with cases where the central orientation is not obvious enough. In spite of that, it presents a low central orientation with a heavy tail in which samples appear likely nearby the limits of distribution compared to others such as normal distribution, as presented in Fig. 2. In the first iterations, the population diversity is increased by developing the scale factor which aims to assure those possible paths of the individuals have stretched/flattened over the search area (i.e., low central orientation). Indeed, the cosine distribution can achieve this objective. In addition, this distribution exhibits a significant abidance through range and limits and therefore is more practicable than intermittent distributions such as quadratic and trigonometric distributions. Hence, the use of cosine distribution is appropriate in cases which are restricted by finite limits and requires a low density around the center (i.e., the samples are scattered more equally). The probability density function (PDF) of the cosine is given by^42,43^:

5 $$ f\left( {y|a,b} \right) = \frac{1}{2b}\cos \left( {\frac{y - a}{b}} \right)\quad y^{min} \le y \le y^{max} $$

where ${\text{y}}^{{\max}}$ and ${\text{y}}^{{\min}}$ are maximum and minimum value of random variables, respectively, ${\text{a}} = \left( {{\text{y}}^{{\max}} + {\text{y}}^{{\min}} } \right)/2$ is the mean (median, location, and mode), and ${\text{b}} = \left( {{\text{y}}^{{\max}} - {\text{y}}^{{\min}} } \right)/{\uppi }$ is scale parameter. The two parameters of the distribution are designed to be completely appropriate in the ambient of the problem of interest. Thus, the output of cosine distribution is limited in the range [−1, 1]. The variance of the proposed cosine distribution is about 0.19 and is calculated as $\left( {y^{max} - y^{min} } \right)^{2} \left( {\pi^{2} - 8} \right)/4\pi^{2}$.

###### The logistic distribution

On the other hand, the logistic density curve is symmetrical, bell-shaped, and has a larger amplitude in the central area of distribution and lighter tails on the limits’ sides compared to the curve of cosine, as shown in Fig. 2. The probability density function (PDF) of the logistic distribution is given by^44,45^:

6 $$ f\left( {y|a,b} \right) = \frac{1}{b}e^{{\left( { - \frac{y - a}{b}} \right)}} \left( {1 + e^{{\left( { - \frac{y - a}{b}} \right)}} } \right)^{ - 2} \quad - \infty < y < \infty $$

This stage involves increasing in exploitation strength whereas preserving some exploration for the problems which offer a large search space at the end of optimization. In the meantime, the location and scale parameters can identify the logistic distribution completely. In consequence, in this work, the scale parameter is set to 0.1 for achieving lighter tail with more exploitation. According to the values drawn from Gaussian distribution in the range [0, 1], the location parameter is shifted from zero to preserve the population diversity and offer adequate exploration capability till the end of the optimization process. The variance of the proposed logistic distribution is about 0.033 which is lesser than the cosine distribution and calculated as b^2 π^2/3.

##### Adaptive crossover operator integrated with scaling factor

The use of low crossover rate or high scale factor produces large disturbances which are beneficial for population diversity but decrease the speed of convergence. The convergence will be rapid, but also premature if the contrary happens, so that, these operators can be valuable in case of integrating^30^. Hence, the crossover probability constant is chosen for linearly increasing across the track of iterations from nearby zero to 0.5, as follows:

7 $$ CR = g/\left( {2G} \right) $$

where $g$ is the generation (iteration /time) and $G$ is the maximum generation. The available parts of the scaling factor is chosen by producing the binary crossover mapping matrix A with size n × d, as follows.

8 $$ A_{ij} = Ind_{ij} \ge CR\quad \forall i \in \left\{ {1, 2, \ldots , n} \right\}, j \in \left\{ {1, 2, \ldots ,d} \right\} $$

where $Ind$ is a random number which is initialized for each *jth* element of the *ith* individual and is produced from the uniform distribution in the range [0, 1]. The above mentioned continuous scaling factor is mapped using the (0–1) matrix A as follows:

9 $$ F_{ij} = F_{ij} \cdot A_{ij} \quad \forall i \in \left\{ {1, 2, \ldots , n} \right\}, j \in \left\{ {1, 2, \ldots ,d} \right\} $$

Observe that if $$ A_{ij} = 1$$, then the scale factor holds its value, else its value is zero. Therefore, as the number of iterations increased, the number of elements that have zero value is increased in the scaling factor matrix in an effort for increasing the exploitation with time.

##### Adaptive elite selection mechanism

Mutations schemes of the MVDE method need a suitable number of chosen individuals to attend as parents in order to lead the search operation. So, the qualified parents of size $$ N_{p}$$, are chosen depending on the top-ranked solutions of the adaptive crossover rate as follows:

10 $$ N_{p} = n \times \left( {1 - CR} \right) $$

Depending on the strategy size, an acceptable number of parents is recognized among the qualified parents. The MVDE algorithm construction retains the ability to integrate and handle five mutation schemes.

#### Multi-variant mutation-crossover process

The time horizon of the optimization process is divided evenly into five successive iterative sub-processes (phases/periods) after initialization. According to choose one of the five kinds of mutation schemes, the population in each phase is modified. During the first iteration, the solutions are updated utilizing the proposed less greedy evolution variants such as (DE/rand/k) to cover the search area and to recognize the encouraging areas by benefiting from their explorative capability. The less greedy variants discover the most encouraging area which is used to lead the search at the end of optimization operation to share more information among the used schemes through the greedy strategies such as (DE/best/k). To focus only on the optimal solution guiding to the desired rapid convergence, the low disturbances of these variants are essential.

According to previous suggestions, the chosen five mutation schemes incorporated with the updated scaling factor (i.e., includes crossover) are applied through mutation-crossover operation, as follows below.

##### Purely explorative stage

Actually, the differences between population individuals are the basis of the disturbance utilized in all variants of the evolution. The “DE/rand/2” variant is the famous least greedy mutation scheme which has a top priority for exploration, and is applied to the first 20% of iterations, as follows:

11 $$ x_{i,j} \left( {g + 1} \right) = x_{{r_{1} ,j}} \left( g \right) + F_{ij} \left( {g + 1} \right).\left( {x_{{r_{2} ,j}} \left( g \right) - x_{{r_{3} ,j}} \left( g \right)} \right) + F_{ij} \left( {g + 1} \right).\left( {x_{{r_{4} ,j}} \left( g \right) - x_{{r_{5} ,j}} \left( g \right)} \right) $$

where $$ r_{1}$$,$$r_{2}$$,$$ r_{3}$$, $$r_{4}$$, and $$r_{5}$$ are five mutually limited random integers chosen from the range [1,$$ N_{p}$$], and all vary from the mutated index $$ i$$.

Two binary scaling factors are produced independently in this variant, therefore each element of the group member $x_{i,j}$ has the chance to search either through one of the parts of the previous equation, by both parts, or remain the same (the later chance increases during optimization progress). Several of these updated chances appear in the following other kinds.

##### More explorative stage

The less greedy and explorative “DE/rand/1” variant is implemented in the next group of iterations as follows:

12 $$ x_{i,j} \left( {g + 1} \right) = x_{{r_{1} ,j}} \left( g \right) + F_{ij} \left( {g + 1} \right).\left( {x_{{r_{2} ,j}} \left( g \right) - x_{{r_{3} ,j}} \left( g \right)} \right) $$

As a result of the existence of a single binary scaling factor, this scheme comprises only two updated chances.

##### Balanced stage

The most appropriate current solution is integrated into the “DE/current-to-best/1” strategy for leading the search to the global optimal with more aesthetic rapid convergence (exploitative conduct). Differences between random solutions are utilized to balance such conduct for updating the robustness as explorative conduct. So, at the middle of the search area, this variant is applied to attain a good balance between exploration and exploitation as follows:

13 $$ x_{i,j} \left( {g + 1} \right) = x_{{r_{1} ,j}} \left( g \right) + F_{ij} \left( {g + 1} \right).\left( {x_{best,j} \left( g \right) - x_{i,j} \left( g \right)} \right) + F_{ij} \left( {g + 1} \right).\left( {x_{{r_{1} ,j}} \left( g \right) - x_{{r_{2} ,j}} \left( g \right)} \right) $$

where $$x_{best}$$ is the best individual over the whole current generation $$ g$$. This scheme holds the four kinds of updated chances revealed in “DE/rand/2”.

##### More exploitative stage

The population in the next sub-domain handled due to the greedy and exploitative “DE/best/2” variant where the four kinds of updated chances are available as follows:

14 $$ x_{i,j} \left( {g + 1} \right) = x_{best,j} \left( g \right) + F_{ij} \left( {g + 1} \right).\left( {x_{{r_{1} ,j}} \left( g \right) - x_{{r_{2} ,j}} \left( g \right)} \right) + F_{ij} \left( {g + 1} \right).\left( {x_{{r_{3} ,j}} \left( g \right) - x_{{r_{4} ,j}} \left( g \right)} \right) $$

##### Purely exploitative stage

At last, the positions of the population are updated by implementing the highly greedy and exploitative “DE/best/1” variant during the last sub-iterations as follows:

15 $$ x_{i,j} \left( {g + 1} \right) = x_{best,j} \left( g \right) + F_{ij} \left( {g + 1} \right).\left( {x_{{r_{1} ,j}} \left( g \right) - x_{{r_{2} ,j}} \left( g \right)} \right) $$

According to the description and difficulty of the problem, the above mentioned mutation schemes can be redescribed or reduced, respectively. At the end of each generation, the mutated individuals compared to the previous population, and the best solutions permitted for surviving to the next generation. We are the original source and the owners of this new algorithm. The original source code is available at (https://www.mathworks.com/matlabcentral/fileexchange/70997-mvde)^46^.

### ANN model

In this work, the optimization of the weights and structure of ANN is regarded by applying MVDE algorithm where each solution in the population of the MVDE holds both weight and structure solution. In this strategy, a specific fitness function which is dependent on the weights and structure of ANN is the base to measure inputs, different weights, number of hidden-layers, and number of nodes in each hidden-layer^47^.

The training phase consists of two main stages: structure optimization and weight update. The network structure is optimized during the training process by selecting important hidden nodes that minimize the output error. The weights of the network are updated by maximizing the diversity of the output from hidden nodes. The weight update stage is proposed to enhance the performance of the structure optimization^48,49^.

- Structure optimization: finding a compact topology with the minimum number of hidden nodes is the goal of the structure optimization stage. This can be achieved by choosing the important nodes from the network while neglecting the reminder.
- Weight update: selecting the most important hidden nodes from the initial network is proposed to produce a compact structure of the network during the structure optimization.

The selected hidden node is then divided into two new hidden nodes which have the same number of weight connections as their parents. The new weight connections are calculated as follows:

16 $$ w_{1} = \left( {1 + \theta } \right) \times w $$

17 $$ w_{2} = - \theta \times w $$

where $w$ is the weight of the existing node, and ${ }w_{1}$ and $w_{2}$ are the weights of the two produced nodes. To avoid a large change in the existing network functionality, the value of $\theta$ should be within small range.

The search process can be accelerated by identifying the suitable number of hidden nodes in ANN architecture.

#### Solution representation

In this work, two one-dimensional vectors are considered for solution representation. One vector describes the structure solution which holds binary values of 0 and 1 while another vector contains weights and biases with real numbers in range [−1, 1]. The first vector has three sections; two sections are for the number of hidden-layers and the number of nodes in each of these layers which occupy three cells each. These three cells also keep the binary values of the number of hidden-layers and the number of nodes in each of these layers^47^.

The feature selection section is added to the structure solution representation for classification since the values of the input nodes, and the number of input nodes is essential which its dimension is equal to the number of features in the dataset. The *ith* feature in the complete feature set is held in the subset of a selected feature if the value of *ith* in this section is equal to 1 while the subset of selected feature does not contain this feature if the cell presents 0 value.

For weights and biases, the second vector contains weights and biases in real values in which the number of them is calculated based on the structure solution^48^.

#### Fitness function

The use of an effective fitness function is necessary to select a solution that minimizes the objective function and to evaluate the quality of it in successive iterations. In this paper, the fitness function is used to minimize the classification error, the number of selected features, and the size of ANN with good generalization capability which is calculated by the average of the error ε, the ration of the selected features, and the ration of the number of weights and biases (connections). The classification error which presents the percentage of misclassified training samples is calculated as follows:

18 $$ E\left( p \right) = \frac{100}{{N_{p} }}\mathop \sum \limits_{i = 1}^{{N_{p} }} (y_{i} - \overline{y}_{i} ) $$

where $n_{p} { }$ is the number of samples, and $y_{i}$ and $\overline{y}_{i}$ are the target and actual output of the network respectively. The second part of the fitness function evaluates the ration of the selected features is as follows:

19 $$ R_{f} = \frac{{n_{s} }}{{n_{f} }} $$

where $n_{s}$ is the number of the selected features and $n_{f}$ is the number of the complete set of features. The third part is the number of connections (weights and biases) evaluated by the fitness function as follows:

20 $$ P_{c} = \frac{1}{{n_{c} }}\mathop \sum \limits_{i = 1}^{{n_{c} }} (w_{i} + b_{i} ) $$

where $n_{c}$ is the total possible number of connections that are utilized by the network, $w_{i}$ is the active number of the weights, and $b_{i}$ is the active number of biases connections. Thus the fitness function $f\left( s \right)$ of the solution s is calculated as follows:

21 $$ f\left( s \right) = E\left( p \right) + a_{1} *R_{f} + a_{2} *P_{c} $$

where $a_{1}$ and $a_{2}$ are user-defined constants within the range of 0 and 1 which are utilized to control the significance of three terns of fitness calculation and they are set to 0.1 in this work.

## Results and discussion

Classification problems are implemented using MATLAB R2017a software on Windows 10 and executed on a PC with an Intel Core i7-5600U processor of 2.6 GHZ 8.0 GB. The maximum number of iterations is set to 100 and the population size is set to 40.

Fifteen classification datasets are evaluated using the MVDE method. The datasets are from different sources^49–52^. Datasets with different issues of instances and attributes are chosen for validating MVDE. Four heuristic algorithms: DE, CSO, WOA, SCA, and PSO algorithms are used to compare and valuate with the MVDE. Table 1 outline the description of the datasets.

**Table 1:** Description of datasets.

Dataset	Number of instances	Number of features	Number of classes
Iris	150	4	3
Australian credit	690	14	2
German credit	1000	24	2
Glass	214	9	6
Hillvalley	606	100	2
Ionosphere	351	34	2
Waveform	5000	40	3
Spambase	4601	57	2
Vehicle	846	18	4
Arrhythmia	452	279	16
Wine	178	13	3
Zoo	101	16	2
Wisconsin Breast Cancer	699	9	2
Heart Statlog	270	13	2
WBCD	569	31	2

The performance of the proposed algorithm and compared methods for finding solutions are measured using eight metric indices. The metric indices are the average of classification accuracy, best, mean, worst, standard deviation (Std), the number of selected features, complexity of the network (active number of connections/total number of connections), and time cost (Seconds) of solutions for denoting the sturdiness and firmness of all implemented methods. The chance to find optimal solutions is increased when candidate solutions broadcast over a wide area of search space. Moreover, the average ranking test is used to judge the performance of MVDE and other methods. The best results for all algorithms using different metric indices are presented in bold.

**Figure 3:**
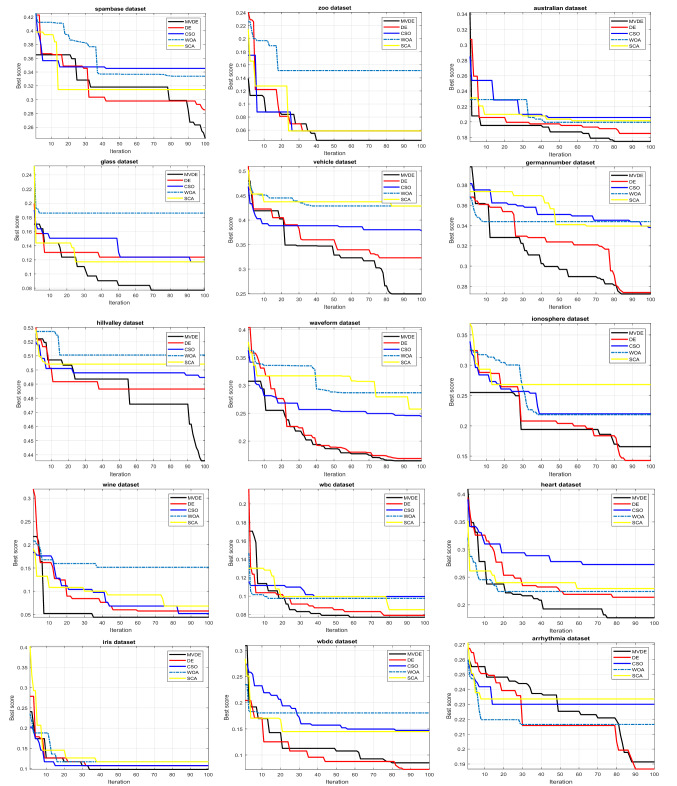
Convergence curves for datasets by different optimizers.

In Fig. 3 which indicates the convergence rates of different methods for various datasets, the MVDE algorithm achieves better performance compared to other methods. The convergence rates of MVDE algorithm are rapid compared to the other algorithms except in case of arrhythmia, ionosphere, and WBCD datasets where DE algorithm is the faster and competes MVDE. Besides, the compared algorithms; CSO, WOA, and SCA contend each other to converge in various benchmarks and converge slowly than MVDE. Mainly, the MVDE is an impactful algorithm to use in various datasets which are utilized in this study.

Table 2 outlines the average classification accuracy which indicates the performance on test data for all datasets using the selected features from different algorithms. We can observe from the table that MVDE is the best performing over all other optimizers except in Australian and zoo datasets where DE is doing better than MVDE. The solutions that have the best average of fitness maximizes the accuracy of the classification and minimizes the selected features’ number is presented in Table 3 which indicates that the best solutions are attained by the MVDE. Considering these results, MVDE has the capability for searching the area of space adaptively outperforms the other algorithms. The average mean and worst solutions are presented in Tables 4 and 5. These results assure the superiority of MVDE over compared algorithms.

**Table 2:** Average classification accuracy of different optimizers.

Dataset	MVDE (%)	DE (%)	CSO (%)	WOA (%)	SCA (%)	PSO (%)
Iris	**95.556**	94.074	95.556	92.593	92.593	87.407
Australian Credit	88.245	**88.406**	86.473	85.99	87.762	76.49
German credit	**73.333**	73.111	71.444	72.333	73	72
Glass	**96.875**	93.229	90.104	93.75	93.229	48.438
Hillvalley	**55.678**	51.832	51.648	51.465	48.901	49.451
Ionosphere	**86.032**	85.714	82.857	76.19	79.365	81.905
Waveform	**85.867**	85.933	82.489	74.733	78.022	80.733
Spambase	**80.507**	77.488	76.014	69.13	69.396	76.329
Vehicle	**75.556**	68.571	68.889	62.54	66.825	41.905
Arrhythmia	**82.843**	80.147	79.167	80.637	78.676	61.029
Wine	**96.855**	96.855	91.195	89.308	95.597	94.969
Zoo	96.667	**97.778**	93.333	76.667	91.111	52.222
Wisconsin Breast Cancer	**96.508**	96.508	93.968	94.286	95.079	94.127
Heart Statlog	**84.362**	83.128	80.658	81.481	78.189	76.543
WBCD	**95.322**	93.567	90.448	86.355	83.821	92.398

**Table 3:** Best fitness function of different optimizers.

Dataset	MVDE	DE	CSO	WOA	SCA	PSO
Iris	**0.097706**	0.097706	0.097706	0.10723	0.10723	**0.097706**
Australian credit	0.17464	0.17257	0.19742	0.19742	0.19949	**0.1498**
German credit	**0.26532**	0.27389	0.30968	0.34397	0.3396	0.30532
Glass	**0.077143**	0.090476	0.097143	0.097143	0.11048	0.61048
Hillvalley	**0.43575**	0.48076	0.46119	0.48287	0.50105	0.47982
Ionosphere	**0.13072**	0.13792	0.18763	0.21843	0.22735	0.16151
Waveform	**0.15814**	0.1606	0.19843	0.25231	0.24529	0.19806
Spambase	**0.23977**	0.25734	0.27371	0.31788	0.31459	0.27031
Vehicle	**0.245**	0.28385	0.31446	0.36791	0.34138	0.64092
Arrhythmia	**0.15896**	0.18663	0.22024	0.21307	0.22366	0.51454
Wine	**0.044316**	0.044316	0.052316	0.057673	0.052316	0.065673
Zoo	**0.044359**	0.044359	0.066607	0.066607	0.044359	0.3632
Wisconsin breast cancer	**0.070926**	0.079106	0.089331	0.085241	0.085241	0.087286
Heart Statlog	**0.1713**	0.1713	0.21433	0.22421	0.2295	0.23604
WBCD	**0.070007**	0.07252	0.10221	0.14303	0.14492	0.10332

**Table 4:** Mean fitness function of different optimizers.

Dataset	MVDE	DE	CSO	WOA	SCA	PSO
Iris	**0.097706**	0.10088	0.10405	0.11358	0.11358	0.11993
Australian credit	**0.17947**	0.18706	0.20294	0.19897	0.20225	0.19552
German credit	**0.27201**	0.27865	0.32775	0.35677	0.34389	0.32579
Glass	**0.088254**	0.10159	0.12381	0.14011	0.12381	0.61492
Hillvalley	**0.46297**	0.48652	0.48431	0.49354	0.50233	0.48287
Ionosphere	**0.14803**	0.15516	0.20863	0.2523	0.24632	0.17183
Waveform	**0.16292**	0.17024	0.2274	0.30933	0.2615	0.22268
Spambase	**0.24966**	0.26909	0.3049	0.34258	0.33572	0.28404
Vehicle	**0.27032**	0.31729	0.34655	0.40043	0.37388	0.64266
Arrhythmia	**0.18092**	0.18713	0.22717	0.22075	0.22955	0.52104
Wine	**0.044316**	0.054102	0.078983	0.092549	0.065649	0.083011
Zoo	**0.044359**	0.047955	0.074695	0.15019	0.04919	0.3632
Wisconsin breast cancer	**0.075016**	0.081151	0.094784	0.094103	0.088649	0.087968
Heart Statlog	**0.17483**	0.18986	0.25522	0.24537	0.23832	0.24142
WBCD	**0.077392**	0.083472	0.11735	0.17577	0.16586	0.11169

**Table 5:** Worst fitness function of different optimizers.

Dataset	MVDE	DE	CSO	WOA	SCA	PSO
Iris	**0.097706**	0.10723	0.10723	0.11675	0.11675	0.14532
Australian credit	**0.18706**	0.20363	0.2057	0.20001	0.2057	0.27041
German credit	**0.27826**	0.28817	0.33817	0.36675	0.34675	0.33675
Glass	**0.097143**	0.12381	0.15714	0.18603	0.14381	0.61714
Hillvalley	**0.48114**	0.49231	0.49703	0.51057	0.50415	0.4853
Ionosphere	**0.16558**	0.18465	0.21984	0.28753	0.268	0.17884
Waveform	**0.16717**	0.18666	0.25702	0.34845	0.27831	0.24607
Spambase	**0.26555**	0.2849	0.3453	0.37594	0.36911	0.29942
Vehicle	**0.31643**	0.345	0.37834	0.42875	0.42867	0.64365
Arrhythmia	0.19239	0.18783	0.23128	0.23272	0.23355	0.52599
Wine	**0.044316**	0.060316	0.11632	0.15166	0.076316	0.10334
Zoo	**0.044359**	0.055147	0.084133	0.19468	0.058852	0.3632
Wisconsin Breast Cancer	**0.077061**	0.083196	0.099556	0.099556	0.093421	0.089331
Heart Statlog	**0.17659**	0.21363	0.27837	0.27183	0.24537	0.24537
WBCD	**0.085083**	0.093273	0.14716	0.20373	0.19769	0.119

Table 6 shows the results of standard deviations for the achieved values of the fitness to investigate the firmness and sturdiness among compared methods with MVDE. From the results, we can observe that the MVDE outperforms the compared methods due to the faintness of the compared methods for exploring and exploiting the search space.

**Table 6:** Standard deviation of different optimizers.

Dataset	MVDE	DE	CSO	WOA	SCA	PSO
Iris	**0.0**	0.0054986	0.0054986	0.0054986	0.0054986	0.023968
Australian Credit	0.0066554	0.015631	0.0047814	**0.001373**	0.0031626	0.065379
German credit	0.0064815	0.0082479	0.015708	0.011648	**0.0037796**	0.017747
Glass	**0.010184**	0.019245	0.030551	0.044518	0.017638	0.003849
Hillvalley	0.02401	0.005773	0.020056	0.01491	**0.0016178**	0.0027902
Ionosphere	**0.017431**	0.025662	0.018201	0.034573	0.02046	0.0091206
Waveform	**0.0045368**	0.014295	0.029303	0.050504	0.016522	0.024031
Spambase	**0.013894**	0.014223	0.036675	0.029979	0.029251	0.014626
Vehicle	0.039991	0.030975	0.031941	**0.030635**	0.047725	0.0015157
Arrhythmia	0.019029	**0.00062295**	0.006037	0.010501	0.0052074	0.0058749
Wine	**8.4984e−18**	0.0085771	0.033307	0.051465	0.01222	0.019013
Zoo	**0.0**	0.0062283	0.0088404	0.072437	0.0083674	**0.0**
Wisconsin Breast Cancer	0.003542	**0.002045**	0.0051464	0.0077422	0.004257	0.0011807
Heart Statlog	**0.0030548**	0.021639	0.035517	0.024246	0.0080821	0.0048312
WBCD	**0.0075424**	0.010424	0.025819	0.03063	0.028016	0.0078903

We can conclude that the MVDE is an outstanding method with various benchmarked problems which attains the best results of different metric indices to avoid trapping in local minima.

The average of selected features are outlined in Table 7 for all compared methods. The number of the selected features is minimized better using the SCA algorithm than others algorithms. Moreover, the MVDE is doing better also and comes in the second place of selecting features number ranking.

**Table 7:** Average selected features of different optimizers.

Dataset	Original features	MVDE	DE	CSO	WOA	SCA	PSO
Iris	4	2	2	2	2	2	2
Australian credit	14	5	5	5	5.3333	5	5.3333
German credit	24	8.3333	8	8.6667	8.3333	8	8
Glass	9	3	3	3	3.6667	3	3
Hillvalley	100	32.6667	35	36.3333	36.3333	30.3333	36
Ionosphere	34	14.6667	12	13.6667	14	11	16.6667
Waveform	40	12.3333	12.6667	15.6667	16	12.3333	18.3333
Spambase	57	21.3333	20.6667	26	20	18.3333	23.6667
Vehicle	18	6.6667	7	9	6.6667	6.3333	7.3333
Arrhythmia	195	81.3333	75.6667	74.3333	62.3333	68.6667	68
Wine	13	4	4.3333	4	5.6667	4	6
Zoo	16	5	5.3333	6	6.6667	5	5
Wisconsin Breast Cancer	9	3	3	3	3	3	3
Heart Statlog	13	4	4.3333	7	4	4	5
WBCD	31	10.6667	11	12.6667	15.3333	10	13.6667

The consuming time is introduced in Table 8. The SCA algorithm executes at the lowest period of time compared to other methods. This means that it is a rapid algorithm to be executed whereas the MVDE, DE, and PSO algorithms are contested to execute in small time. On the other hand, CSO and WOA are high consuming time.

**Table 8:** Execution time in seconds of different optimizers.

Dataset	MVDE	DE	CSO	WOA	SCA	PSO
Iris	1.28	1.28	6.44	1.46	**0.82**	1.50
Australian credit	9.07	8.17	51.15	4.93	**2.16**	9.58
German credit	14.51	12.20	121.49	15.10	**6.47**	12.78
Glass	2.08	2.03	12.73	1.94	**1.14**	1.94
Hillvalley	49.20	58.81	571.89	679.02	**16.32**	50.96
Ionosphere	9.43	9.50	84.13	12.26	**2.22**	8.04
Waveform	99.82	115.73	1278.06	108.78	**20.61**	124.62
Spambase	146.18	169.16	2344.56	243.35	**32.25**	208.75
Vehicle	8.92	7.81	63.74	8.20	**2.28**	7.37
Arrhythmia	192.56	190.88	2015.80	17,241.83	**64.17**	126.34
Wine	1.85	2.14	14.15	2.55	**0.97**	3.01
Zoo	1.66	1.53	10.31	1.17	**0.75**	1.36
Wisconsin Breast Cancer	5.36	5.20	35.18	4.37	**1.87**	6.29
Heart Statlog	3.25	3.13	21.45	2.37	**1.28**	3.15
WBCD	11.40	11.39	108.58	11.34	**3.06**	12.13

In the case of the complexity of the network, Table 9 outlines the complex network of different optimizers. The results show that MVDE and SCA methods outperform in using the least connections of the network. Therefore, MVDE has the ability for training the ANN using a fewer complexity of the network model by a high accuracy.

**Table 9:** Complexity of the network for different optimizers.

Dataset	MVDE	DE	CSO	WOA	SCA	PSO
Iris	0.3818	0.3818	0.3818	0.3818	0.3818	0.3818
Australian Credit	0.1677	0.1677	0.1677	0.1677	0.1677	0.1677
German credit	0.1341	0.1341	0.1341	0.1341	0.1341	0.1341
Glass	0.1714	0.1714	0.1714	0.1714	0.1714	0.1714
Hillvalley	0.0953	0.1282	0.0953	0.1503	0.0953	0.1353
Ionosphere	0.1871	0.1412	0.2125	0.1871	0.1207	0.2125
Waveform	0.1366	0.1031	0.1748	0.1552	0.1031	0.1957
Spambase	0.1593	0.2031	0.1459	0.1593	0.1208	0.2521
Vehicle	0.1417	0.1417	0.4049	0.1835	0.1417	0.1417
Arrhythmia	0.2022	0.1799	0.1757	0.1174	0.0974	0.1072
Wine	0.1355	0.1921	0.1355	0.4212	0.1355	0.2586
Zoo	0.1311	0.1311	0.1311	0.1311	0.1311	0.1311
Wisconsin Breast Cancer	0.1714	0.1714	0.1714	0.1714	0.1714	0.1714
Heart Statlog	0.1355	0.1355	0.1921	0.2586	0.1355	0.2586
WBCD	0.1442	0.1442	0.1216	0.1442	0.1216	0.2236

In a conclusion, the judgment on the different optimizers are presented in Table 10 to rank and order every compared algorithm. From this table, the MVDE algorithm is graded in the first place for all metric indices excluding the average number of selected features, consuming time, and complexity of the network.

**Table 10:** Average of the ranks for different optimizers.

Algorithm	Rank 1	Rank 2	Rank 3	Rank 4	Rank 5	Rank 6	Rank 7	Rank 8	Average rank	Final rank
MVDE	1.3	1.366667	1	1.066667	2.5	3.066667	3.566667	3.3	2.145834	1
DE	2.033333	2.1	2.133333	2.166667	3.033333	3.3	3.233333	3.4	2.675	2
CSO	3.833333	3.7	4.166667	4.1	4.566667	4.266667	5.866667	3.533333	4.254167	5
WOA	4.5	4.766667	5.166667	5.2	4.766667	4.133333	3.466667	4.1	4.5125	6
SCA	4.333333	4.833333	4.4	4.3	3.233333	2	1	2.366667	3.308333	3
PSO	5	4.233333	4.133333	4.166667	2.9	4.233333	3.7	4.3	4.083333	4

Table 11 indicates the results using KNN classifier, which MVDE average classification accuracy results using KNN classifier are better than using ANN classifier in some datasets and vice versa. The values’ range of average fitness, mean, worst, standard deviation are different using KNN classifier than using ANN classifier due to the differences between the two classifiers. Execution time using KNN classifier is more than using ANN classifier, which means that KNN classifier is a time consuming method compared to ANN classifier. Some of classification methods just work with some of the data or applications better than others.

**Table 11:** Average values of different metrics using KNN classifier for MVDE.

Dataset	Classification accuracy (%)	Best fitness	Mean fitness	Worst function	Std	Number of features	Execution time
Australian credit	**88.937**	**0.71071**	**0.71243**	0.7151	0.00236	5	78.63
German credit	78.567	**0.7528**	0.75819	0.7655	0.00656	8	92.50
Hillvalley	59.944	0.8583	0.8624	0.86455	0.00357	**30**	77.44
Ionosphere	**93.81**	**0.6738**	**0.67602**	0.6790	0.00272	11	50.04
Waveform	**84.473**	**0.7106**	**0.71188**	**0.7130**	0.00121	**12**	880.13
Arrhythmia	56.889	**0.8856**	0.88744	0.8891	0.00175	**59**	82.86
Wisconsin Breast Cancer	97.647	0.6623	0.66234	0.6623	**0.0000**	3	65.58
Heart Statlog	**86.543**	**0.7039**	**0.70458**	**0.7058**	**0.00106**	4	51.20

To authenticate the performance of the MVDE technique, the comparison among the MVDE and the lately four published algorithms by other researchers is presented in Table 12^23,24,27–30^. Eleven datasets that are common among this study and the compared studies in the recent researches. The results due to the comparison among the MVDE and the methods in the literature review indicate that the MVDE outperforms the other four methods on iris, Australian credit, German credit, hillvalley, and waveform datasets. In contrast, a slight bad performance of classification on ionosphere, vehicle, wine, zoo, WBC, and heart datasets. For the heart benchmark, MVDE attains well performance of classification over one method, but worse than the other one. On the other side, the MVDE chooses a fewer number of features compared to all other algorithms for all benchmarks. However, it selects a slight more selected features’ number on ionosphere dataset than only one method. In addition, the computational time that is achieved by MVDE algorithm is typically shorter for all datasets except for WBC dataset since the number of selected features is smaller. Additionally, the results validate that the MVDE algorithm’s performance is competitive to the state-of-the art algorithms. MVDE benefits from the advantages of DE algorithm in addition to the modifications applied to it for overcoming DE drawbacks. MVDE is a parameter free optimization method with a high performance and applicable to deal with complex high-dimensional problems. The design of MVDE algorithm with multi variant mutation and adaptive scaling factor help it to select the optimal number of features used for classification which has an impact on the accuracy of the classification.

**Table 12:** Comparison with other methods.

Dataset	Method	Classifier	Ave no. of features	Ave accuracy	Std	Time (s)
Iris	Shunmugapriya et al.^23^	WEKA 3.6.3	2	95.1%	–	2.04
	MVDE	ANN	2	95.556%	0.0	1.28
Australian Credit	Peng et al.^27^	SVM	8.2	87.3%	–	–
	MVDE	ANN	5	88.245%	0.0066554	9.07
German credit	Hancer et al.^24^	KNN	9.13	70.1%	–	–
	Peng et al.^27^	SVM	12.3	70.33%	–	–
	MVDE	ANN	8.3333	73.333%	0.0064815	14.51
Hillvalley	Hancer et al.^24^	KNN	44.96	54.92%	–	–
	MVDE	ANN	32.6667	55.678%	0.02401	49.20
Ionosphere	Hancer et al.^24^	KNN	11.53	91.74%	–	–
	Peng et al.^27^	SVM	16.1	96.6%	–	–
	Mafarja et al.^28^	KNN	16.4	89.9%	0.007	2.899
	Zawbaa et al.^29^	KNN	–	83.6%	–	–
	MVDE	ANN	14.6667	86.032%	0.017431	9.43
Waveform	Mafarja et al.^28^	KNN	26.233	73.7%	0.003	123.546
	Zawbaa et al.^29^	KNN	–	83.6%	–	–
	MVDE	ANN	12.3333	85.867%	0.0045368	99.82
Vehicle	Hancer et al.^24^	KNN	7.73	77.88%	–	–
	MVDE	ANN	6.6667	75.556%	0.039991	8.92
Wine	Mafarja et al.^28^	KNN	8.8	98.9%	0.0	2.492
	Zawbaa et al.^29^	KNN	–	95.3%	–	–
	MVDE	ANN	4	96.855%	8.4984e−18	1.85
Zoo	Mafarja et al.^28^	KNN	9.167	99.3%	0.09	2.485
	Zawbaa et al.^29^	KNN	–	84.6%	–	–
	MVDE	ANN	5	96.667%	0.0	1.66
Wisconsin Breast Cancer	Shunmugapriya et al.^23^	WEKA 3.6.3	3	99.07%	–	7.09
	Mafarja et al.^28^	KNN	5	98%	0.001	3.537
	Zawbaa et al.^29^	KNN	9	95.7%	–	–
	Li et al.^30^	BABC	14.9	92.16%	0.0249	–
	MVDE	ANN	3	96.508%	0.003542	5.36
Heart Statlog	Shunmugapriya et al.^23^	WEKA 3.6.3	4	84.51%	–	11.43
	Mafarja et al.^28^	KNN	8.4	83.3%	0.004	2.803
	Zawbaa et al.^29^	KNN	13	82.2%	–	–
	MVDE	ANN	4	84.362%	0.0030548	3.25

Note: unavailable data are denoted as “–”.

## Conclusion

This work presents a novel optimization algorithm (MVDE), which has multi variant mutation with adaptive scaling factor is developed by integrating adaptive crossover rate with mutation factors and adaptive selection of parent to achieve better performance. The performance of the MVDE algorithm is verified using fifteen real-world problems to ensure its stability, quality, and simplicity. In this paper, the performance and the complexity of the ANN for training process are optimized simultaneously. Different weights, biases, number of nodes of the hidden layers, and selecting inputs during the search process have the opportunity to be checked by providing this method. So, an effective model of ANN with low complexity and classification error has the chance to be found. The goal challenge to balance between exploration (diversification) and exploitation (intensification) is achieved utilizing the MVDE and a varied population is preserved through iterations.

The evaluation is implemented using a set of evaluation criteria to evaluate different aspects of the MVDE algorithm. In addition, DE, CSO, WOA, and SCA methods are applied to solve the problems and to compare the results to MVDE method. The results of the methods in the literature review and the MVDE algorithm are compared to evaluate the performances.

The investigative results infer that the MVDE optimization method is a useful and an appropriate technique to classify data and can move to a particular group of benchmarks. The results also investigate the ability of the MVDE algorithm for dodging the local minima better than the compared DE, CSO, WOA, and SCA methods. The performance of the selected features is promising and better for the features selected by the MVDE. Furthermore, the superiority of the MVDE performance is obviously detected for training ANNs in terms of evaluation metrics.
